# Immune response and protective efficacy of an experimentally developed inactivated oil adjuvant *Brucella abortus* vaccine in BALB/c mice

**DOI:** 10.5455/javar.2024.k841

**Published:** 2024-12-27

**Authors:** Md. Zaminur Rahman, Md. Ariful Islam, Palash Bose, Mst. Minara Khatun, Rokshana Parvin

**Affiliations:** 1 Department of Microbiology and Hygiene, Bangladesh Agricultural University, Mymensingh, Bangladesh; 2 Department of Pathology, Bangladesh Agricultural University, Mymensingh, Bangladesh

**Keywords:** Inactivated oil adjuvant *B. abortus* vaccine, BALB/c mice, Immune response, Protective efficacy

## Abstract

**Objectives:**

This study evaluated the immunogenicity and protective efficacy of an inactivated oil adjuvant *B. abortus* vaccine in BALB/c mice.

**Materials and Methods:**

Mice in group A (*n = *30) received subcutaneous (s.c.) immunization with 0.1 ml of vaccine (1.5 × 10^7^ inactivated *B. abortus *biovar 3 per mouse) and were boosted 4 weeks later. Group B (*n = *30) received normal saline as unvaccinated controls. BALB/c, vaccinated and unvaccinated mice were challenged with *B. abortus* biovar 3 (3 × 10^7^ cells per mouse) at 6 weeks post-vaccination (WPV). Serum antibody levels were assessed at 0, 1, 2, 3, 4, 5, and 6 WPV using RBPT and i-ELISA. Cellular-mediated immune (CMI) response was evaluated by measuring the skin thickness of vaccinated mice’s left and right hind footpads sensitized with *B. abortus* soluble antigen and PBS, respectively. Bacterial persistence and spleen histopathological lesions were evaluated at 1, 2, and 3 weeks post-challenge.

**Results:**

The vaccinated mice developed *B. abortus*-specific serum IgG response from 2 WPV. The highest serum IgG titer was observed in 5-6 WPV (*p* < 0.001). The skin thickness was significantly higher in the left footpad than the right footpad (*p* < 0.001). Huge cellular infiltration with mononuclear and polynuclear cells was noticed in the dermis and sub-dermis areas of the left footpad. The spleen weight and bacterial load in the spleen were significantly reduced in vaccinated mice compared to unvaccinated control mice (*p* < 0.001).

**Conclusions:**

The inactivated oil adjuvant *B. abortus *vaccine induced both humoral and CMI responses, which conferred protection in BALB/c mice against virulent challenge infections.

## Introduction

Brucellosis is a major zoonotic bacterial disease caused by the *Brucella *spp., an important human and animal health problem worldwide. Five species of Brucella—*B. suis* (pigs), *B. ovis* (sheep), *B. melitensis* (goats), *B. abortus* (cattle), and *B. canis* (dogs)—are known to infect domesticated animals. Within *B. abortus*, biovars 1, 2, 3, 4, 5, 6, 7, and 9 have been identified.

Abortion, infertility, retention of the placenta, endometritis, repeat breeding, and hygroma in cattle are caused by *B. abortus*. *B. abortus* can infect goats, sheep, and buffalo in addition to cattle. In Africa, Asia, the Middle East, and Latin America, bovine brucellosis is endemic [1].

Humans can contract brucellosis from animals by handling cattle aborted material, consuming unpasteurized milk, and coming into close contact with diseased animals. Every year, over 5,00,000 cases of human brucellosis are reported worldwide [2]. Human brucellosis is often found in the Middle East, Asia, Central America, Africa, the Indian subcontinent, and Mediterranean nations. Those who work with livestock, butchers, milkers, artificial inseminators, and veterinarians are at risk for brucellosis.

There have been reports of *B. abortus* infections in humans in Bangladesh. Most people in Bangladesh purchase and consume unpasteurized raw milk, which can expose humans to *B. abortus *[3]. Research in Bangladesh’s Sylhet area found that the prevalence of brucellosis was 31.3% among slaughterhouse employees, 52.6% among abattoir employees, 61.1% among dairy farm employees, and 50% among veterinarians [4]. If brucellosis is controlled in animals, human brucellosis can be prevented.

The livestock industry suffers significant financial losses as a result of brucellosis because of abortion, infertility, reduced milk production, and treatment expenses. According to an Indian study, brucellosis causes 3.4 billion USD in economic losses in the livestock sector each year [5]. According to a study, bovine brucellosis costs Brazil 2.10 USD per animal [5].

Brucellosis has become endemic in domesticated animals of Bangladesh. *B. abortus* was isolated from milk, as well as cattle giving birth to an aborted calf. According to a sero-prevalence report, the prevalence of brucellosis in Bangladesh was 3.7% in cattle, 4% in buffalo, 3.6% in goats, and 7.3% in sheep [6]. A study conducted in the Chittagong municipality in Bangladesh recorded a 21.5% prevalence of brucellosis in cattle [7]. Bovine brucellosis causes huge economic losses in dairy farms in Bangladesh [7].

Vaccination, test and slaughter, and quarantine are some of the classic prevention and control measures of bovine brucellosis. In middle- to low-income nations, the test-and-slaughter approach of brucellosis control is not economically viable [5]. One of the best ways to prevent brucellosis in animals is by vaccination. Both live and killed vaccines have been used to control brucellosis in large and small ruminants. Live attenuated Brucella vaccines, such as *B. melitensis* strain Rev. 1, *B. abortus *strain S19, and *B. abortus *RB51, are used in many nations to prevent brucellosis in cattle and small ruminants. The whole-cell bacteria or lysed bacterial cells having intact cell surface structures are used to produce inactivated *B. abortus* vaccine. In some European countries, the inactivated oil adjuvant *B. abortus *biovar 1 was used to treat bovine brucellosis [8]. However, the duration of immunity of killed *B. abortus *vaccine is short and needs booster vaccination.

Live attenuated *B. abortus *vaccines have several problems, including the potential to infect humans if administered to them accidentally while immunizing cattle and the ability to cause abortion in pregnant animals [8]. A live attenuated strain of *B. abortus *can be shed from vaccinated animals, which can then be transmitted to humans via their milk, urine, sperm, or feces [9]. When live *B. abortus* vaccines are administered to calves, the animals may carry *B. abortus *for the rest of their lives. Adult immunization with live attenuated *B. abortus *vaccine in bovines produces orchitis in males [10]. Live *B. abortus *vaccines need a continuous cold chain during transport and storage. Thus, there is a need to develop *B. abortus *vaccines that are effective and safe and can be stored at room temperature. Inactivated *B. abortus* vaccine could be a suitable alternative to live vaccine since it does not cause infection in humans and abortion in pregnant animals and can be stored at room temperature [10]. Developing an inactivated oil adjuvant *B. abortus* vaccine, assessing the vaccine’s protective effectiveness in immunized BALB/c mice after challenge infection with virulent *B. abortus* biovar 3, and determining humoral and cellular-mediated immune (CMI) responses were the three main goals of the current study.

## Materials and Methods

### Ethical approval

As required by the University’s strict bio-safety regulations, animal studies were carried out in an authorized facility. With consent given in 2020 (permit number: AWEEC/BAU/2020/36), the investigations were carried out in compliance with the rules and regulations established by the Bangladesh Agricultural University’s Animal Welfare and Experimental Ethics Committee (AWEEC) in Mymensingh, Bangladesh.

### BALB/c mice

The Department of Microbiology and Hygiene at Bangladesh Agricultural University, Mymensingh, provided the primary animal breeding facilities from which the female BALB/c mice (6–8 weeks old) were obtained. Mice were housed in a climate-controlled environment, and a veterinarian regularly monitored their health conditions. The mice were given unlimited access to tap water and commercial pellet food.

### Bacterial strain

Laboratory stock of *B. abortus* biovar 3 (GenBank accession number: BAU21/S4023) that has been genetically characterized and isolated from a dairy cow [11] was used for the preparation of experimentally developed inactivated oil adjuvant *B. abortus* vaccine. Laboratory stock culture of *B. abortus* biovar 3 was cultured by streaking onto blood agar and *Brucella* selective agar (BSA) (HiMedia, Mumbai, India). The culture was then incubated for five to seven days at 37°C in an incubator with 5% CO_2_. Bacterial colonies grown on BSA were harvested using a sterilized loop. Harvested bacterial colonies were placed into a 15 ml sterile graduated centrifuge tube (Falcon, Birmingham, UK) containing 10 ml normal saline (NS) and mixed thoroughly by vortexing. At 4°C, this bacterial suspension was centrifuged for 30 minutes at 3500 rpm. After being discarded, the supernatant was re-suspended in NS to its initial volume. This step was repeated 3 times. The McFarland turbidity standard 0.5 or 1 (Remel, Kansas, USA) was used to adjust the final washed bacterial cell concentrations to 1.5 × 10^8^ CFU/ml and 3 × 10^8^ CFU/ml. The AMOS-ERY PCR assay was used to confirm that *B. abortus* biovar 3 [11].

### Inactivation of B. abortus biovar 3

*B. abortus* biovar 3 (3 × 10^8^ CFU/ml) was inactivated by 1% formalin (Merck, Darmstadt, Germany) using the procedure outlined by Grilló et al. [12]. Briefly, cell suspension that had been formalin-treated was centrifuged at 15000 rpm for 20 min after being held at 4°C for 72 h in an orbital shaker. The pellet was collected in 5 ml of NS, and the washing procedure was carried out 3 times. Then NS was added to the pellet until there were roughly 3 × 10^8^ inactivated cells/ml. Inactivated *B. abortus* biovar 3 antigen was kept at 4°C until use.

### Purity and sterility test

Gram stain was used to determine the purity of formalin-inactivated *B. abortus* biovar 3. To assess sterility, inactivated antigen was streaked onto a blood agar plate and incubated for 2 weeks at 37°C in an incubator with 5% CO₂_. _The absence of bacterial growth in the blood agar indicated complete inactivation.

### Oil adjuvant

Sterilized mineral oil (HiMedia Mumbai, India) was added with the inactivated *B. abortus *antigen suspension. A commercial blender (Jaipan, Mumbai, India) was used to combine the antigen and mineral oil adjuvant suspension (1:1) with 6% sterilized lanolin (HiMedia, Mumbai, India) [13] and centrifuge it for 20 min at 18000 rpm.

### Safety test

Five BALB/c mice were given a 0.5 ml inactivated oil adjuvant *B. abortus* vaccine subcutaneously (s.c.). The mice were then monitored for 7 days to see if any clinical symptoms, including fever, weakness, and anorexia, developed. Mice were sacrificed by CO_2_ asphyxiation following cervical dislocation. Necropsy of mice was performed at 1 WPV. Spleens of mice were collected aseptically and macerated in PBS using glass pestle tissue grinders (PYREX™ Glass, Thermo Fisher Scientific, Waltham, MA). Blood agar was cultivated with macerated 10% spleen homogenate and incubated for seven days at 37°C with 5% CO_2_. The absence of clinical signs and no growth of *B. abortus* on blood agar from spleen homogenate indicated that the vaccination was safe to use.

### Vaccination and challenge infection

Between the ages of 6 and 8 weeks, 60 female BALB/c mice were randomly assigned to groups A (*n =* 30) and B (*n =* 30). Mice in group A received 0.1 ml (1.5 × 10^7^ inactivated cells per mouse) of inactivated oil adjuvant *B. abortus *vaccine s.c. mice in group B received only 0.1 ml of NS as an unvaccinated control. The dose of 0.1 ml (1.5 × 10^7^ inactivated cells per mouse) was carefully selected based on a combination of previous immunogenicity research in the mice model [14]. Four weeks after the initial vaccination, a booster dose of the vaccine was given. At 6 WPV, both vaccinated and unvaccinated control BALB/c were challenged i.p. with 0.2 ml (3 × 10^7^ cells per mouse) of virulent *B. abortus* biovar 3. Mice were observed and examined for 3 WPC for mortality and clinical illness.

### Rose Bengal plate test

Serum IgG titers of five randomly selected vaccinated mice at 0, 1, 2, 3, 4, 5, 6, and 7 WPV were measured by RBPT according to the method of Zhu et al. [15]. Test sera were diluted two-fold: 1:2, 1:4, 1:8, 1:16, 1:32, and 1:64 using PBS. A white tile with a 1.5 cm diameter circle was used to hold the serially diluted serum (30 µl). Next, 30 µl of Rose Bengal Antigen (ID. vet Rose Bengal, Grabels, France) was added to the serum. Gentle agitation was used to properly combine the serum and antigen. The plate was then examined visually within 4 min for agglutination reaction.

### Indirect enzyme-linked immunosorbent assay

In accordance with the “manufacturer’s instructions,” sera were extracted from five randomly chosen mice at 0, 1, 2, 3, 4, 5, 6, and 7 WPV and examined for an IgG response specific to *B. abortus *using a commercial i-ELISA kit (ID ScreenR Brucellosis serum Indirect Multi-species, Grabels, France). Briefly, all reagents and test serum samples were allowed to reach room temperature (RT) (21 ± 5°C). The microtiter plate wells were filled with 190 µl of dilution buffer 2. Every microtiter plate well, except the positive and negative control wells, received a duplicate of 10 µl of test serum. Each well was rinsed and cleaned three times using 300 µl of wash solution following a 45 min incubation period at room temperature. After that, 100 µl of conjugate was added, and the mixture was incubated for up to 30 min at room temperature. After emptying the wells, 300 µl of wash solution was used three times. Each well received a single addition of 100 µl of substrate solutions, which were then incubated for 15 min at room temperature in a dark setting. The addition of 100 µl of stop solution halted the reaction.

Test sera’s optical density (OD) value was measured at 450 nm absorbance using an ELISA reader (Stat Fax^®^ 4200, Florida, USA). Standard deviation (SD) ± mean OD was used to express the results.

### Delayed-type hypersensitivity skin test

The CMI response in vaccinated mice was evaluated using the delayed-type hypersensitivity (DTH) skin test [16]. At 5 WPV, the left hind footpads of mice (*n = *5) received intradermally 20 µl PBS containing 20 µg of *B. abortus *soluble antigen (BASA), and the right hind footpads were intradermally injected with 20 µl PBS (negative control). The BASA was prepared according to the previously described method [16]. After injection, digital slide calipers (SKU: 17839, China) were used to measure the thickness of the right and left footpads every 24 h, 48 h, and 72 h. Footpad swelling differences of ≥ 0.2 mm were considered a positive CMI response.

### Histopathological evaluation of skin test

Histopathological changes of footpads of vaccinated mice were evaluated at 72 h post-injection with BASA. Five mice were sacrificed by CO_2_ asphyxiation followed by cervical dislocation. A transverse cut made with a razor blade to the distal tibia/fibula separated the mouse’s hind footpads. The footpads were fixed in 10% neutral buffered formalin after being longitudinally sectioned. After cutting the formalin-fixed tissue samples into pieces that were 2–3 mm thick, they were thoroughly cleaned with water, dehydrated in increasing alcohol grades, and then cleared in xylene. Blocks of paraffin were used to implant the desiccated tissues. Following the normal procedure, the blocks were further processed to create tissue slices (5 µm) and stained with hematoxylin and eosin (H&E) stain [17]. An Olympus CX43 light microscope with an EP50 camera (Olympus Corporation, Japan) was used to view the dyed slides. Histopathological changes were assessed and analyzed between the left and right footpads.

### Bacterial persistence in the spleen

Vaccinated (*n = *5) and unvaccinated (*n = *5) mice were sacrificed by CO_2_ asphyxiation followed by cervical dislocation at 1, 2, and 3 WPC. Mice were necropsied in a class II biosafety cabinet (Thermo Scientific, Waltham, MA, USA) of type A2. Using sterile rat tooth forceps, spleens were separated, defatted, weighed, and split into two equal sections. One part was used for bacteriological examination and another for histopathological examination. The splenic part was placed in a glass pestle tissue grinder (PYREX™ Glass, Thermo Scientific, Waltham, MA, USA) and macerated to prepare a 10% tissue homogenate in PBS. The spleen tissue homogenate (1 ml) was diluted 10 times, and a duplicate (0.1 ml) was streaked onto blood agar plates from each dilution. For three to five days, inoculated plates were incubated at 37°C with 5% CO_2_. The number of bacterial colonies on each plate was between 30 and 300 CFU. After counting, the CFU was represented as log_10_ CFU/gm of the spleen.

### Spleen histopathology

Half a portion of the defatted spleens was fixed in 10% neutral buffer formalin. After cutting the formalin-fixed spleen tissue into pieces that were 2-3 mm thick, it was thoroughly cleaned with water, dehydrated in increasing alcohol grades, and then cleared in xylene. Using a microtome (ModelRM2245, Leica Biosystems, Wetzlar, Germany), the dehydrated tissues were serially sectioned at a thickness of 4 µm after being embedded in paraffin blocks. H&E stain was used to stain all the tissue sections. Histopathological changes were assessed and analyzed between vaccinated and unvaccinated groups [15].

### Analysis of statistics

To determine if there was a significant difference between groups A and B in terms of IgG titers, footpad skin thickness, spleen weight, and bacterial persistence in the spleen, the Student’s t-test was employed. All analyses were conducted using computer software (IBM SPSS version 27, California, USA). A *p*-value of less than 0.05 was deemed statistically significant.

## RESULTS

### Confirmation of B. abortus biovar 3

**Figure 1.**
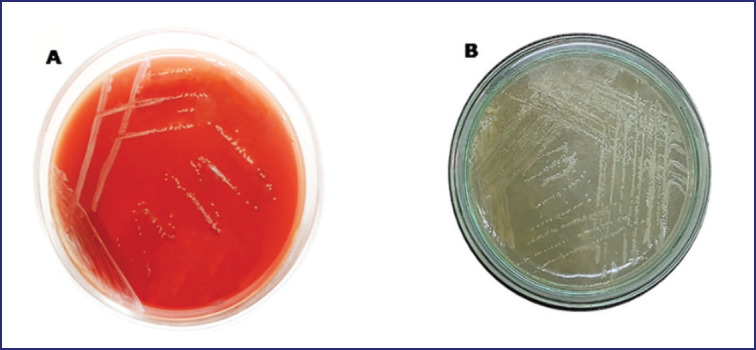
The *Brucella abortus *biovar 3 Colony on blood agar shows non-hemolytic whitish colonies (A). In BSA, *B. abortus* biovar 3 produces small translucent, honey-colored dew drop-like colonies (B).

**Figure 2.**
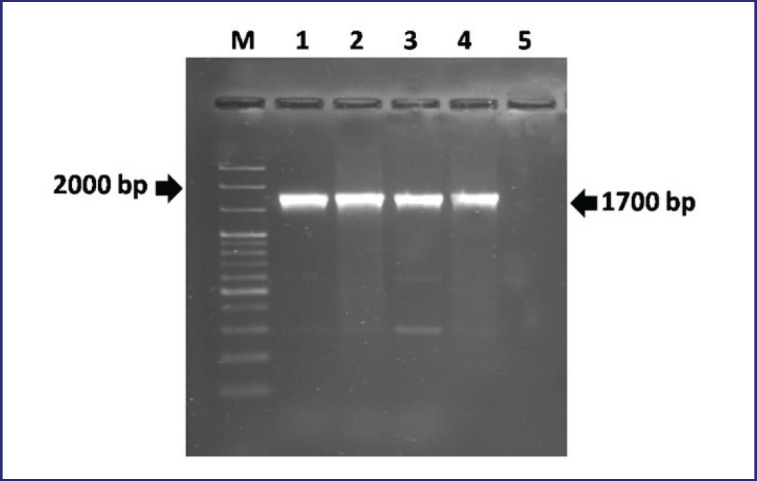
Molecular detection of *B. abortus* biovar 3 by AMOS-ERY PCR assay. Lane M: I kb DNA ladder (Thermo Scientific, USA), Lane 1-3: DNA extracted from bacterial colonies obtained from blood agar and *Brucella* selective agar, Lane 4: Positive control, Lane 5: Negative control.

Blood agar-revived colonies of *B. abortus* biovar 3 were white-gray, glossy, round, convex, and nonhemolytic (Fig. 1A). A small, pinpoint-sized colony with a honey color was seen on the BSA (Fig. 1B). The AMOS-ERY PCR assay effectively produced a 1700 bp PCR amplicon of a DNA region associated with the *ery *(erythritol) gene of *B. abortus* biovar 3 (Fig. 2).

### Safety of vaccine

Up to seven days after receiving the *B. abortus* vaccine, BALB/c mice did not exhibit any clinical signs of disease. The body temperature of the vaccinated mice was in the normal range (36.5 ± 0.5°C). No mortality was observed in vaccinated mice. *B. abortus* isolates were not recovered from the immunized mice.

### Antibody response in mice measured by RBPT

Table 1 displays the antibody response data in the serum of the mice that received the vaccination. Between 2 and 6 WPV, RBPT was able to identify the *B. abortus*-specific serum antibody response in vaccinated mice. All mice were RBPT positive at 6 WPV. The highest antibody titer was found at 5 (1:16) and 6 (1:16) WPV. Antibody response in the serum of mice without vaccine had a negative RBPT result.

### Measurement of serum IgG in vaccinated mice by ELISA

**Figure 3.**
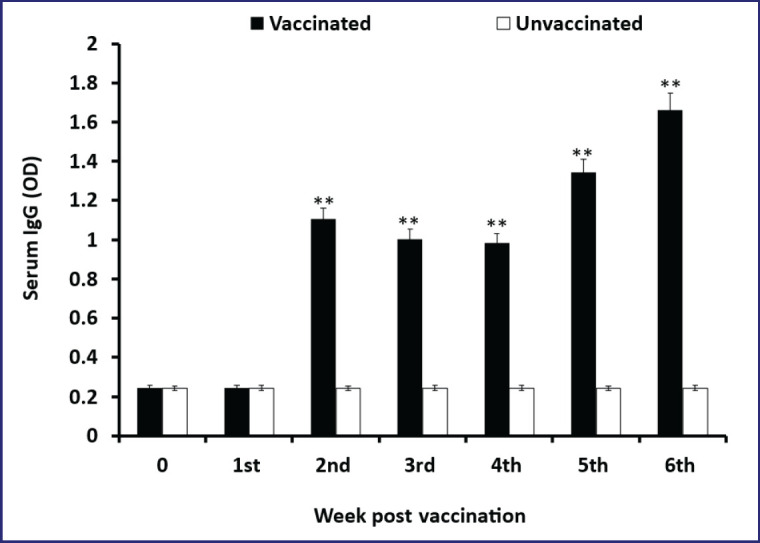
*Brucella abortus *specific serum IgG in vaccinated and unvaccinated control mice at 1st, 2nd, 3rd, 4th, 5th, and 6th WPV measured by indirect ELISA. Data represent the mean of five mice at each time point, and error bars represent the standard deviations (SD). Statistically significant differences between vaccinated and unvaccinated control groups are indicated by asterisks (**p < 0.001).

In the 1st, 2nd, 3rd, 4th, 5th, and 6th WPV, the mean ELISA OD values of the sera from the vaccinated mice were 0.245 ± 0.003, 1.1056 ± 0.038, 1.0028 ± 0.009, 1.042 ± 0.006, 1.3436 ± 0.091, and 1.663 ± 0.088, respectively (Figure 3). The IgG responses first appeared in the serum of immunized mice at the 2nd WPV. It progressively rose, reaching the highest production level at the 5th and 6th WPV. In mice that were not immunized, the serum antibody response was 0.202 ± 0.001. Antibody levels were statistically significant (*p *< 0.05) higher in vaccinated mice than in unvaccinated control mice.

### Cell-mediated immune response measurement

Table 2 displays the results of the DTH skin test. The skin thickness was significantly higher in the left footpad than in the right footpad at 24, 48, and 72 h post-tests.

### Necropsy evaluation of footpads

**Figure 4.**
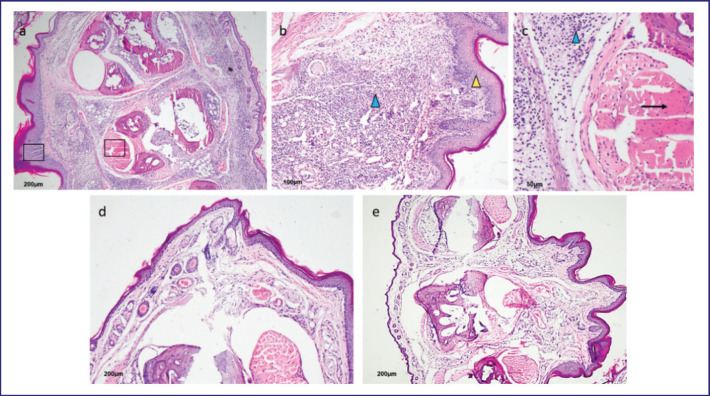
Histopathological lesions of vaccinated mice’s left and right hind footpads at 72 h following intradermal inoculation with BASA and PBS, respectively. The left footpad epithelium was strongly swollen, and tissue reactions were prominent at 72 h post-inoculation. (a) A huge cellular infiltration and the beginning of tissue necrosis were explicitly strong at the base of the footpad, where antigen was injected. (b) Epidermis is highly edematous (yellow arrowhead) with huge cellular infiltration (blue arrowhead). Necrosis of the muscle fibers was prominent (black arrow). The black box in Figure 4a indicated a higher magnification of that selected region, which can be seen in the following 4b and 4c, respectively. d&e) the right footpad (PBS control) and afresh footpad control showed no swelling, minimum infiltration, intact muscle tissue, and absence of edema. The bar indicates the scale.

Vaccinated mice that received BASA revealed necrotic areas in the peri-muscular sub-dermic conjunctive tissue of the left footpad (Fig. 4a). Thickening of the epidermis with edema, polymorphonuclear neutrophils, diffuse mononuclear infiltration, and arteritis were also observed (Fig. 4b). In the dermis, diffuse mononuclear cell infiltration and accumulation with muscular degeneration were observed (Fig. 4c). The right footpad of mice showed mild perivascular and sub-dermal mononuclear cell infiltration. The lack of involvement of muscular tissue and the absence of inflammatory infiltrations in the dermis showed the normal thickness of the right footpad (Fig. 4d) compared to the left footpad. Fresh control mice (neither injected with BASA nor immunized with inactivated oil adjuvant *B. abortus *vaccine represent similar lesions (Fig. 4e) as do the right footpad of mice.

**Table 1.** RBPT test results of vaccinated mice.

WPV	Sera tested (*n)*	RBPT positive sera *n (%)*	RBPT positive sera at titer *N (%)*		
			1:4	1:8	1:16
1	5	0(0)	0(0)	0(0)	0(0)
2	5	3(60)	3(100)	0(0)	0(0)
3	5	3(60)	3(100)	0(0)	0(0)
4	5	4(80)	4(100)	4(100)	0(0)
5	5	4(80)	4(100)	4(100)	4(100)
6	5	5(100)	5(100)	5(100)	5(100)

WPV: week post-vaccination, RBPT: rose Bengal plate test.

**Table 2.** DTH skin test results in vaccinated mice after intradermal injection of left and right footpads with *B. abortus* soluble antigen (BASA) and PBS, respectively.

HPI	Footpad swelling (mean ± SD)		Differences (a-b) (mm)	*p*-value
	Left footpad (mm) a	Right footpad (mm) b		
0	2.8 ± 0.120	2.8 ± 0.190	0	1
24	4.8 ± 0.091	3.5 ± 0.120	1.3	< 0.001
48	4.3 ± 0.079	3.2 ± 0.040	1.1	< 0.001
72	4.5 ± 0.063	3.2 ± 0.082	1.3	< 0.001

HPI: hours post-injection, mm: millimeter, SD: standard deviation.

### Spleen weight

Table 3 displays the findings of the spleen weight of vaccinated and unvaccinated control mice. At 1, 2, and 3 WPC, the weight of the spleens in the vaccinated mice was 0.452 ± 0.013 gm, 0.430 ± 0.039 gm, and 0.410 ± 0.017 gm, respectively. The unvaccinated control group’s spleen weight was substantially greater at 1, 2, and 3 WPC (0.522 ± 0.041 gm, 0.570 ± 0.019 gm, and 0.690 ± 0.021 gm, respectively) than the vaccinated groups (*p* < 0.05 or *p* < 0.001).

### Persistence of bacteria in the spleen

Table 4 displays the bacterial burdens in the spleen of the mice that received the inactivated oil adjuvant vaccination and the unvaccinated control groups. The vaccine group’s spleen had bacterial burdens of log_10_ 5.64 ± 0.41 CFU/gm, log_10_ 3.5 ± 0.05 CFU/gm, and log_10_ 0.4 ± 1.10 CFU/gm at 1, 2, and 3 WPC. However, the unvaccinated control group’s bacterial burden was considerably higher than the vaccinated group’s at 1, 2, and 3 WPC (*p* < 0.001).

### Histopathological findings in the spleen

**Figure 5.**
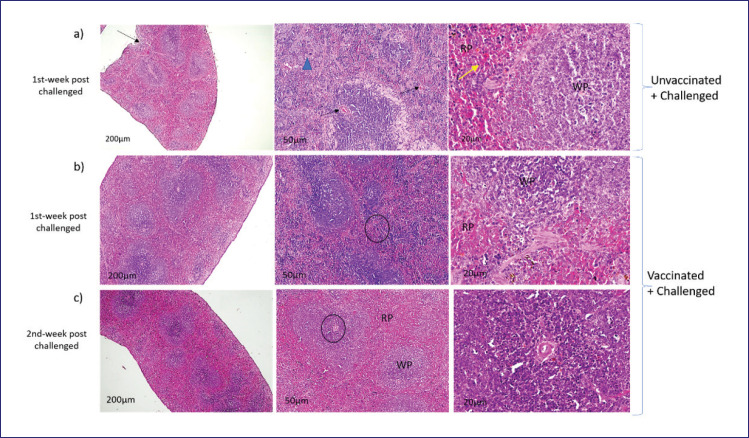
Morphologic changes in the spleen after being challenged with *B. abortus* biovar 3 in both vaccinated and unvaccinated mice. (a) Unvaccinated but challenged mice showed significant tissue reactions in the spleen. Thickened capsule (black arrow), apoptosis or necrosis of white pulp (blue arrowhead), and destroyed red blood cells (yellow arrow) were prominent. (b) Minimum reaction in the red pulp (RP) and white pulp (WP). (c) The spleen is almost healthy at 2 weeks post-challenge. Round black circles in b and c indicate the selected regions magnified in the next right image.

Figure 5 illustrates the histopathologic analysis of the spleen to examine the pathological impact of the virulent *B. abortus* biovar 3 strains in mice that were vaccinated with oil adjuvant and those that were not. The unvaccinated mice’s spleen showed a poorly defined border between the red and white pulp. The destruction of red blood cells and diffuse infiltration of inflammatory cells occurred throughout the spleen. The vaccinated mice challenged with *B. abortus* biovar 3 had a distinct red-white pulp boundary, mild hemolysis, and more inflammatory cells in the red pulp (RP), although the spleen was less damaged. The unvaccinated control mice’s tissue sections revealed splenitis along with a noticeable swelling of the capsule. Congestion causes the blood vessels in the capsule to enlarge and become noticeable. The white pulp showed signs of lymphoid depletion, apoptosis, and necrosis in the lymphocytes. The spleen structure of the *B. abortus* biovar 3-vaccinated mice remained normal with clear lymphocyte differentiation.

**Table 3.** Spleen weight of vaccinated and control BALB/c mice following challenge with *B. abortus *biovar 3.

WPC	Spleen weight (gm) mean ± SD		Weight reduction (gm) (a-b)	*p*-value
	Vaccinated mice b	Unvaccinated mice a		
0	0.318 ± 0.014	0.317 ± 0.008	−0.001	0.808
1	0.452 ± 0.013	0.522 ± 0.041	0.07	< 0.05
2	0.430 ± 0.039	0.570 ± 0.019	0.14	< 0.05
3	0.410 ± 0.017	0.690 ± 0.021	0.28	< 0.001

WPC: week post-challenge, SD: standard deviation.

**Table 4.** Bacterial load in spleen after challenged with *B. abortus* biovar 3.

WPC	Bacterial load in spleen (Log_10_ CFU/gm)		Log reduction	*p*-value
	Vaccinated mice	Unvaccinated control mice		
1	5.6 ± 0.41	9.1 ± 0.70	3.5	< 0.001
2	3.5 ± 0.05	10.8 ± 0.53	7.3	< 0.001
3	0.4 ± 1.10	11.3 ± 0.11	10.9	< 0.001

WPC: week post-challenge, CFU: colony forming unit.

## Discussion

Vaccination continues to be the primary goal of the brucellosis control program since it is the most efficient method for decreasing disease burden. Its advantages include preventing brucellosis, controlling bacterial shedding, stopping animal-to-animal transmission, and reducing the spread of pathogens from animals to people. [18]. There is still no ideal vaccine for brucellosis. A new search for a brucellosis vaccination is ongoing [9,10]. Live vaccines, currently used to prevent brucellosis, have numerous disadvantages and might not be appropriate for nations with few resources since they require a cold chain for transport and storage [19]. The ideal vaccine choice in underdeveloped nations may be safe to deliver to animals and can be maintained at room temperature. The inactivated *B. abortus* vaccine is safe for pregnant animals and can be stored at room temperature. There is no risk of human infection from accidentally administering an inactivated Brucella vaccine. Therefore, the effectiveness of an inactivated oil adjuvant *B. abortus* vaccine in protecting mice from a virulent *B. abortus* infection was assessed in this work.

Over time, numerous vaccinations have been created to defend against brucellosis. Brucella vaccines that are inactivated include *B. melitensis* H38 and *B. abortus *strain 45/20 [9]. *B. melitensis* H38 was tested on mice and cows, whereas *B. abortus *strain 45/20 was employed on cattle and sheep. These inactivated Brucella vaccines induced persistent antibody titers in vaccinated animals [9]. The inactivated oil adjuvant *B. abortus* vaccine was prepared in the current study using *B. abortus *biovar 3, which was obtained from dairy cattle [6]. *B. abortus *biovar 3 has already been genetically characterized [11]. *B. abortus* biovar 3 was molecularly validated using the AMOS-ERY PCR assay, and the findings concurred with those of Islam et al. [11]. Since BALB/c mice are a widely used animal model for studying *Brucella* vaccines, the preclinical trials of the experimentally generated vaccine were conducted on these animals [20]. Due to the absence of *B. abortus* biovar 3 isolation from the spleen of vaccinated mice and the absence of clinical signs, the experimentally developed inactivated oil adjuvant *B. abortus *vaccine was shown to be safe in BALB/c mice.

Mineral oil is recognized as an effective adjuvant. It causes irritation and tissue damage at the injection site and generates damage-associated molecular patterns (DAMPs). Antigen-presenting cells like dendritic cells and macrophages are drawn to tissue injury. Mineral oil adjuvant was used to prepare livestock and poultry vaccines. Lanolin was used as an emulsifier to increase the stability of the inactivated oil adjuvant *B. abortus* vaccine.

The doses and *B. abortus* biovar 3 strains utilized as challenges in the vaccination experiment are important. When over 99% of challenge controls contract an infection, the optimal circumstance has been obtained [21]. In the current investigation, the BALB/c mouse was experimentally challenged with 3 × 10^7^ CFU/ml *B. abortus *biovar 3. It was found that every control mouse who hadn’t received a vaccination got the infection.

Both humoral and CMI play a role in protection against infection caused by *B. abortus*. However, CMI is most important in protecting against infection since *B. abortus* is an intracellular pathogen. In this investigation, RBPT was used to quantify the antibody-mediated humoral immune response in BALB/c mice that had received vaccinations. In cattle, this test is quite sensitive, particularly when it comes to identifying antibodies produced by the *B. abortus *S19 vaccination. At 2, 3, 4, 5, and 6 WPV, the serum of vaccinated mice showed the antibody response. The immunization of water buffaloes with *B. abortus *S19 vaccine produced antibodies detected by RBPT at 1, 2, 4, and 6 WPV [22]. In this study, at 2 WPV, 60% of mice showed seropositivity in RBPT. Immunization of guinea pigs with subunit Brucella vaccine caused 70% seropositivity at 2 WPV [23]. This study observed the highest RBPT antibody titer (1:16) in the BALB/c mice at the 5 and 6 WPV, which might have resulted from booster vaccination. BALB/c mice vaccinated with *B. suis* strain 2 showed peak serum IgG titer at 3 WPV [15].

 In this study, five vaccinated BALB/c mice’s pooled sera were used to analyze the humoral immune response’s kinetics using i-ELISA. The serum IgG in BALB/c mice induced from 2 to 6 WPV by oil adjuvant *B. abortus *vaccine as determined by i-ELISA. The serum IgG was highest at 5 and 6 WPV following booster vaccination. The vaccinated group’s IgG OD value was significantly greater (*p* < 0.05) than that of the unvaccinated control group. The maximum OD unit of IgG was seen at 4 and 5 WPV in Swiss Albino mice who received the recombinant outer member protein vaccination [24]. In guinea pigs, Abd El-Tawab et al. [25] examined the humoral immune reactions to the inactivated oil adjuvant *B. melitensis* vaccination. Their findings concur with those of the present research.

The DTH skin test measures CMI response in mice [20]. The skin thickness of the mice’s footpads was dramatically affected by the intradermal antigen injection in this investigation; at 24 h, 48 h, and 72 h following the antigen injection, the left footpad of immunized mice had considerably greater values than the right footpad (*p* < 0.001). Following 72 h after sensitization, Pasquevich et al. [26] observed a substantial increase in footpad thickness in mice immunized with Brucella species outer member protein (U-Omp16 or U-Omp19) in comparison to controls that were given PBS. The significant difference in skin thickness between the immunized groups and the control negative group in guinea pigs was also noted by Yingst et al. [27]. During this investigation, the histology of the right and left footpads of control mice that had received a vaccination and those that hadn’t were also compared. The left footpad showed necrotic areas in the peri-muscular sub-dermic conjunctive tissue, polymorphonuclear neutrophils, and diffuse mononuclear infiltration and arteritis. Portella et al. [28] have found similar histological findings. The results of this study’s DTH skin test demonstrated that experimentally developed inactivated *B. abortus* vaccination can elicit a CMI response.

By comparing the spleen weight, bacterial load in the spleen, and histopathological lesions of the spleen between vaccinated and unvaccinated control mice after virulent challenge infection with *B. abortus *biovar 3, the protective effectiveness of the inactivated oil adjuvant *B. abortus *vaccine was assessed. When compared to vaccinated mice, the unvaccinated control mice’s spleen weight was considerably higher (*p* < 0.05). The pro-inflammatory effects of *B. abortus* LPS may be the cause of the unvaccinated mice’s higher splenic weight [10]. He et al. have obtained similar results [14]. The *B. abortus *vaccine protects BALB/c mice, as evidenced by the significantly decreased (*p *< 0.05) bacterial colonization in vaccinated mice when compared to unvaccinated control mice. Vaccinated mice’s spleens displayed distinct lymphocyte differentiation and normal splenic structure. Significant capsule thickening and splenitis were noted in the unvaccinated mice. Zhu et al. [15] observed similar histological findings. The spleens of vaccinated mice underwent no significant alterations, indicating that the inactivated *B. abortus* vaccine confers protection in the BALB/c mice. To prevent infection from intracellular microorganisms, the IgG plays a crucial function.

The IgG enhances the phagocytosis process via opsonization. The significant increase of DTH reaction of vaccinating mice indicating Th1 cell-mediated immune response was produced by the inactivated oil adjuvant *B. abortus* vaccine [29].After mice were challenged with virulent *B. abortus *biovar 3 strains, the experimentally created inactivated oil adjuvant *B. abortus *vaccination provided 100% protection. In guinea pigs challenged with *B. abortus* 544, the *B. abortus *S19 vaccination provided 84% protection [25].

## Conclusion

According to the results of this investigation, BALB/c mice responded favorably to the newly developed inactivated oil adjuvant *B. abortus* biovar 3 vaccination regarding humoral and CMI responses. The experimentally developed inactivated oil adjuvant *B. abortus *vaccination for cattle is currently undergoing field trials.

## List of abbreviations

BASA, *B. abortus* soluble antigen; BSA, Brucella selective agar; CFU, Colony forming unit; CMI, Cell-mediated immunity; DTH, Delayed type hypersensitivity; h, hour(s); H & E, Hematoxylin and Eosin; i-ELISA, Indirect enzyme-linked immunosorbent assay; i.p., Intraperitoneally; min, Minute(s); NS: Normal saline; OD, Optical density; PBS, Phosphate buffered saline; PCR, Polymerase chain reaction; RT, Room temperature; s.c., Subcutaneously; SD, Standard deviation; RBPT, Rose Bengal plate Test; USD, United States dollar; WPC, Week post-challenge; WPV, Week post-vaccination.
